# Household Hints and Recipes

**Published:** 1874-07

**Authors:** 


### Household Hints & Recipes.

To Keep off Flies.—Oil of cloves is said to be very effectual in protecting animals against flies and mosquitoes. A few drops rubbed on the skin are sufficient for a whole day.—*Southern Medical Record.*

Dandruff.—Make a lather of carbolic-acid soap, such as is used for the teeth, and once a week give the head a thorough shampooing with it; wash the head clean after this with warm water.

Limpid and Flexible Varnish.—Anhydrous alumina stearate, dissolved in turpentine, is the article recommended for this purpose, and it is said to be unalterable at elevated temperatures.

For Colic in a Filly.—Dr. Horne gives the following as his remedy: One quarter-ounce of prepared chalk in her food every morning for a few days, to correct the acidity of the bowels, which causes it.—*M. Farmer.*

Keep Away Mice.—To keep seeds from the depredations of mice, mix some pieces of camphor gum with the seeds. Camphor placed in drawers or trunks will prevent mice from doing them injury.

To Cure a Balky Horse.—Simply place your hand over the horse's nose, and shut off his wind until he wants to go, and then let him go. So says one that has long tried it. The remedy is simple and merciful.

Lotion for Fetid Feet.—Permanganate of potash fifteen parts, distilled water 1,000 parts. The feet to be washed twice a day with the lotion. They are then to be carefully dried, and powdered either with potato-starch or lycopodium.

Copying Ink.—No press is needed, but simply lay a damp sheet of copying paper over the writing and rub with a paper knife; mix thirty grains of extract of longwood, seven grains crystal soda, half an ounce of water. Boil in an enameled saucepan till desolved; then, while stirring well, add thirty grains of glycerine, one grain of chromate of potash, previously dissolved in a few drops of water and four grains powdered gum arabic.—*Ex.*

Preserving Fence Posts.—Stir into boiled linseed oil, pulverized charcoal to the consistency of thick paint; put a heavy coat of this over well-seasoned posts and no man will live long enough to see them rot: expense per post, two cents.—*Ex.*

Mocking-Bird Food.

| | | |
|---|---|---|
| Hemp seed | . . | 1 pound. |
| Rice | . . . | 3 ounces. |
| Buttered crackers, powered | | 8 " |

Grind the hemp-seed and rice to a fine powder, add the crackers, a little lard, and mix well.

Cure for Scratches. —"I directed my man," says the *Maryland Farmer*, "to keep the parts affected washed clean, and, in addition, to wash it with a solution of chloride of lime—about a good teaspoonful dissolved in a teacup of water. In three days the disease was about cured.

To Remove Garlic from Milk.—A correspondent of the *American Agriculturist* says that wood charcoal is an excellent absorbent of the disagreeable flavor in milk. He uses it every spring by dropping a piece three or four inches long and two inches thick into each pan of milk.

Iced Apples.—Pare, core and slice apples of a large, tart kind. Bake them till nearly done. Put them away to get entirely cold; then prepare some sugar icing, and first pouring off all the juice, lay the icing thickly on the tops and sides, as much as you can. Return them to the oven to just harden and be set. Serve with cream.

Lime Water for Wasp Stings.—Dr. Danverne writes to a French journal that some time ago he was stung on the head and face by a number of wasps. The pain was great, and he had no ammonia at hand, nor was there a druggist near by. Recollecting the fact that lime was good for burns, it occurred to him to try it for the relief of the burning sensation produced by the stings. It answered the purpose perfectly, and he has since advised its use in some twenty cases of wasp stings, and it has always caused an instant cessation of the pain. The remedy is a simple one, and worth "making a note of."

Horses Overheated.—The Secretary of the Society for Prevention of Cruelty to Animals recommends the following preparation for animals suffering from being overheated: To one pint of water put one ounce of chloride of ammonia, one ounce sweet spirits of nitre, one drachm of tinct. aconite; give a tablespoonful every hour or two.

Distemper Cure.—A correspondent of the Cincinnati *Gazette* cures the distemper in horses, he claims, with the following: Take sassafras root, wild cherry bark and burdock root, a quart of each kind; put in a kettle with two gallons of water, boil down to one gallon, give one quart twice a day, in bran and oats. Continue the above until cured.

Killing Lice on Cattle.—"I never found any remedy that I like," says the *Maryland Farmer*, "so well as anquintum." A piece about the size of a common pea thoroughly rubbed on the head at the roots of the horns, will effectually do the business. Care should be taken not to let the animal get wet for forty-eight hours after the application, and there is no danger.

How to Tell Counterfeits.—To discover spurious greenbacks or national bank-notes, divide the last two figures of the number of the bill by four, and if one remains the letter on the genuine will be A; if two remains it will be B; if three, C; and should there be no remainder, the letter will be D. For example, a note is registered 2,461; divide sixty-one by four and there will be one remaining. According to the rule the letter on the note will be A. In case the rule fails, be certain that the note is counterfeit.—*Ex.*

Worcestershire Sauce.—A correspondent of *The Garden* gives a recipe for this popular sauce. He says: "I do not offer it as Lea & Perrin's, but I do say that it is equal, if not superior to it, in my opinion. It is not a recipe copied out of a book, but one I have known a long time. Half a pound shallots; 1 oz. pimento, powdered; ½ oz. mace, powdered; ¼ oz. cayenne; half a nutmeg, powdered; ¼ ℔. anchovy fish; 1 oz. salt; 3 pints vinegar; 6 oz. soy. Chop or bruise the shallots, beat up the anchovy fish, mix all together, let it stand for a month or two, and lastly strain through a coarse sieve."

Lotion of Acetic Acid for— Baldness. The following lotion is superior for a shampooing liquid, for removing dandruff, and as a useful and pleasant application for baldness. It is, of course, moderately stimulating, and in those cases in which the hair follicles are not destroyed, but have become merely inactive, it is likely to prove efficacious. Take of

| | |
|---|---|
| Acetic acid, . . | 1 drachm. |
| Cologne water, . . | 1 ounce. |
| Water, to make in all, . | 6 ounces. M. |

Kalsomining Fluid.—The following is commended as a good kalsomining fluid for walls:

| | |
|---|---|
| White glue, . . . | 1 pound. |
| White zinc, . . . . | 10 pounds. |
| Paris white, . . . | 5 " |
| Water, . . . . | sufficient. |

Soak the glue over night in three quarts of water, then add as much water again, and heat on a water bath till the glue is dissolved. In another pail put the two powders and pour on hot water, stirring all the time, until the liquid appears like thick milk. Mingle the two liquids together, stir thoroughly, and apply to the wall with a whitewash brush.

Milk Diet in Dysentery.—We have had occasion more than once to refer to the value of a milk diet in certain cases, as attested by the experience of eminent English physicians. We may add the testimony of Dr. Barret, who states in the *Archivee de Medecine Navale*, that he has made use of milk in chronic dysentery among soldiers and sailors returning from China. He considers a milk diet superior to all other treatment in such cases. The milk must be pure, unmixed with water, as fresh as possible, and not boiled. Sufficient milk was given to a patient, but nothing else allowed to pass his lips. Diarrhœa, if it appears, lasts but a few days. No change of diet is to be made, and no medicine given. If the physician fears the persistence of the diarrhœa, a small quantity of bismuth may be prescribed. If the milk pass through the bowels undigested, pepsin will remedy the defect in the digestive process. After a time the fæces become solid, the patient thinks himself cured, and craves other food. This is the dangerous period, for too early relaxation of the diet may cause a relapse. White of eggs, rice, cream, and the lightest possible things are to be admitted sparingly; and when the patient feels convalescent, and will endure the restrictions no longer, he is to return by the slowest degrees to his former diet.

Ether Glue.—An excellent liquid glue is made by dissolving glue in nitric ether. The ether will dissolve only a certain amount of glue, consequently the solution cannot be made too thick. The glue thus made is about the consistency of molasses, and has double the tenacity of that made with hot water. If a few bits of india-rubber, cut into scraps the size of buck-shot, be added, and the solution be allowed to stand a few days, being stirred frequently, it will be all the better, and will resist the dampness twice as well as glue made with water.

---

Cleansing Bottles, Etc.—Bottles that have had medicines in them may be cleansed by putting ashes in each, immersing them in cold water, and then heating the water gradually till it boils. After boiling an hour, let them remain in the water till it is cold. Wash them in soapsuds, and rinse them till clear in fair water. Pie-plates that have been long used for baking are apt to impart an unpleasant taste on account of the rancidity of the butter and lard imbibed. Put them in a brass kettle, with ashes and cold water, and boil them an hour.

---

Roup Remedy.—Though I may be unable to advance anything new in regard to a remedy for the roup, still, there may be at this time many new subscribers seeking for information which my method may benefit. My plan is to take four or five drops of solution of chlorinated soda in a teaspoonful of cold water, and turn down the throat of the fowl or chick, while another person holds its bill open. Also to wash the bill and nostrils thoroughly (if the whole head gets soaked so much the better) in warm water, containing a few drops of the solution. It is perfectly harmless, and I have never failed to cure, though I sometimes have to administer two or three times a day for a number of days. It is well to bathe the head in clear water after, to remove the solution from the eyes of the bird.—*Poultry Bulletin.*

---

How to Cure Stammering.—No stammering person ever found any difficulty in singing. The reason of this is, that by observing the measure of the music—by keeping time—the organs of speech are kept in such position that enunciation is easy. Apply the same rule to reading or speech, and the same result will follow. Let the stammerer take a sentence, say this one—"Leander swam the Hellespont"—and pronounce it by syllables-scan it, keeping time with his finger if necessary, letting each syllable occupy the same time, thus, Le-an-der-swam-the-Hel-les-pont, and he will not stammer. Pronounce slowly at first, then faster, but still keep time; keeping time with words instead of syllables. Practise this in reading and conversation until the habit is broken up. Perseverance and attention is all that is necessary to perform a perfect cure.

---

Fly Paper.—As "fly-time" returns again, our readers may be looking for means of getting rid of the troublesome insects. The following are approved recipes for making "fly papers," taken from the *Druggists' Circular:*

Dip filtering or bibulous paper in one of these solutions:

1. Quassia chips, . . 1 ounce.
   Water, . . . . 1 pint.

Boil ten minutes and strain. Some add one drachm of powdered nux vomica, and boil with the quassia.

2. Black pepper, . . 1 ounce.
   Boiling water, . . . ½ pint.

Make an infusion and strain.

3. Arseniate of soda, . . 10 grains.
   Water, . . . 4 ounces.

Dissolve. The paper is to be simply immersed in the liquid, and dried. When wanted for use, a piece of the paper is laid in a plate with a little sweetened water. The formula No. 3 is the surest, but requires caution in using.

---

Bottled Ginger Beer.—This is also a seasonable recipe, and one which affords a harmless and refreshing substitute for beverages that owe their stimulating properties to alcohol. It is made thus: Lump sugar, 1 ℔.; good unbleached Jamaica ginger (well bruised), 1 oz.; cream of tartar, ¾ oz. (or tartaric acid, ½ oz.); 2 or 3 lemons (sliced); boiling water, 1 gal. Macerate with frequent stirring, in a covered vessel, until barely lukewarm, then add of yeast 1½ or 2 oz. (about two-thirds of a wineglassful), and keep it in a moderately warm place to excite brisk fermentation; the next day rack or decant the liquor, and strain it through a jelly-bag or flannel; allow it to work for another day or two, according to the weather; then skim it, again decant or strain, and put it into bottles, the corks of which should be "wired" down.

